# Osteoarthritis Progression: Mitigation and Rehabilitation Strategies

**DOI:** 10.3389/fresc.2021.724052

**Published:** 2021-08-23

**Authors:** Devin Drummer, Jeremy McAdam, Regina Seay, Arny Ferrando, S. Louis Bridges, Jasvinder A. Singh, Marcas Bamman

**Affiliations:** ^1^UAB Center for Exercise Medicine, University of Alabama at Birmingham, Birmingham, AL, United States; ^2^Department of Cell, Developmental, and Integrative Biology, University of Alabama at Birmingham, Birmingham, AL, United States; ^3^Department of Geriatrics and Center for Translational Research in Aging and Longevity, University of Arkansas for Medical Sciences, Little Rock, AR, United States; ^4^Department of Medicine, Hospital for Special Surgery, New York, NY, United States; ^5^Division of Rheumatology, Weill Cornell Medical Center, New York, NY, United States; ^6^Division of Clinical Immunology and Rheumatology, University of Alabama at Birmingham, Birmingham, AL, United States; ^7^Department of Epidemiology, University of Alabama at Birmingham, Birmingham, AL, United States; ^8^Veterans Affairs Medical Center, Birmingham, AL, United States; ^9^Florida Institute for Human and Machine Cognition, Pensacola, FL, United States

**Keywords:** osteoarthritis, rehabilitation, exercise, therapeutics, total joint arthroplasty

## Abstract

Osteoarthritis is the most common form of arthritis and is a substantial burden for patients with the disease. Currently, there is no cure for osteoarthritis, but many emerging therapies have been developed to aid in the mitigation of disease progression. When osteoarthritis reaches the end-stage of disease many patients undergo total joint arthroplasty to improve quality of life, yet some experience persistent pain and mobility limitations for extended periods following surgery. This review highlights recent therapeutic advancements in osteoarthritis treatment consisting of pharmacologics, nutraceuticals, biologics, and exercise while emphasizing the current state of post-arthroplasty rehabilitation.

## Introduction

Osteoarthritis (OA) is a disease of high research priority as tens of millions of people in the US experience its deleterious impact on mobility and quality of life. The pathogenic process in OA is distinct from other musculoskeletal aging diseases such as osteoporosis (1), and consists of the breakdown of articular cartilage in a diseased joint and compromises periarticular structures (2). While more common in the elderly and in women (3), OA does occur in younger adults and is not sex specific (4). Overall, incidence of OA has been increasing and is projected to grow to over 100 million diagnosed cases worldwide by 2050 (5). Additionally, OA causes significant economic burden, as illustrated using data from the 2015 Medical Expenditure Panel Survey which highlighted an annual excess national healthcare cost in the United States of $45 billion for patients with OA (6). Due to these projections, preventative interventions and mitigation strategies for the disease have been developed. Prior to 2017, many reviews summarized the state of the field concerning OA and potential interventions such as exercise (7–9). One such review was written by Pedersen and Saltin, two pioneers in the muscle biology field, highlighted exercise as medicine for OA and suggested possible mechanisms for how exercise acts as an analgesic and attenuates inflammatory mediators of the disease (10). However, a recent commentary from our group highlighted the unsettling reality of high incidence of inactivity among individuals with OA even in light of the preponderance of evidence supporting the benefits of exercise on OA pathology and symptomology (9). This prior review discussed what was known at the time with respect to exercise medicine in OA and highlighted potential avenues for significant scientific breakthroughs in the OA field. The current mini-review aims to highlight the strides made in recent years, as well as summarize the current state of knowledge on the molecular underpinnings of OA and how the periarticular physiology seems to both contribute and be impacted, particularly in the context of recovery following total joint arthroplasty (TJA)—a definitive surgical treatment for end-stage OA. The literature search consisted of probing PubMed with search terms coupled with osteoarthritis such as: etiology, rehabilitation, exercise, treatments, nutraceuticals, and total joint arthroplasty with an emphasis on human literature since 2015. Any omitted topics were due to space constraints, and do not reflect a lack of importance of the research.

### Etiology of OA

OA is the most common joint disease, characterized by the failure of the joint as an organ (11). Knee OA is the largest culprit of physical disability in the world (5, 12). Many factors contribute to the pathogenesis of OA, which is defined as the maladaptation of a joint and its periarticular structures (2). Lifestyle factors such as previous connective tissue injuries, obesity (13), and advancing age (2), appear to increase the likelihood of symptomatic OA. Until recently, OA was generally considered a non-inflammatory disorder; however, there is growing evidence that inflammation contributes significantly to OA (14), and that inflammation is a key regulator of osteoarthritis pathogenesis (15). Furthermore, it appears that tissues in direct contact with the arthritic joint may be susceptible to overt pro-inflammatory signaling (2, 16). Specifically, signal transduction pathways such as tumor necrosis-factor like weak inducer of apoptosis (TWEAK) and receptor fibroblast inducible factor 14 (Fn14), tumor necrosis-factor alpha (TNF-α) and receptor (TNF-αR), as well as Interleukin-6 (IL-6) and receptor (IL-6R) are implicated in chondrocyte senescence and dysregulation of periarticular tissues such as skeletal muscle through activation of the transcription factors nuclear factor kappa-light-chain-enhancer of activated B cells (NFκB) and signal transducer and activator of transcription 3 (STAT3) (16, 17). Such data highlight the possibility for OA biomarkers that could be used to guide treatment that optimizes the mitigation of disease progression.

Further interrogation of potential biomarkers of OA have largely been viewed from the context of chondrocytes (18–22), the cells responsible for cartilage formation. Their distinct phenotype is influenced by the state of the joint as a whole and they secrete numerous factors to regulate joint health (18). Enzymes such as A Disintegrin and Metalloproteinase with Thrombospondin motifs (ADAMTS) possess anabolic and catabolic roles in cartilage with high expression of ADAMTS-7 and ADAMTS-12 being implicated in OA pathogenesis (23). Furthermore, proteins such as chitinase-3-like-protein-1 (CHI3L1), a synovial fluid and serum marker of OA, is linked to matrix metalloproteinase (MMP) activity as well as inflammatory interleukin signaling (18). Other markers of OA progression such as cartilage acidic protein-1 (CRTAC1), high temperature requirement A serine peptidase-1 (HTRA1), gremlin-1 and others have been identified using proteomic methods (18). In some instances, it is necessary to probe the chondrocytes or chondrocyte explants with common *in vivo* drivers of OA such as IL-1β to elicit a divergence in OA chondrocyte secretion of inflammatory mediators (e.g., cytokines and chemokines) relative to healthy cells (18). Furthermore, increased disease severity appears to drive greater inflammatory signaling such as expression of the transcription factor NFκB and proteolytic enzyme MMP-13, both associated with cartilage degradation (19). Using logistical regression Liu et al. report that exercise may mitigate some of these deleterious signaling events (19). SIRT1 decline has been linked to MMP activity and OA, but when elevated due to exercise, SIRT1 appears protective for chondrocytes and hyaline cartilage (20, 22, 24). Therefore, inflammation seems to be a key mechanism of OA pathogenesis, but exercise may be a potential means to overcome this deleterious signaling.

While chondrocytes have been heavily studied as a source of potential OA biomarkers, the other periarticular tissues may provide additional insight necessary to distinguish an OA signature vs. other conditions such as aging or osteoporosis (21). In a recent review, Henrotin et al. suggested that well-designed ‘omics studies, or multiplexing of known biomarkers such as Coll2-1 and PIIANP for collagen breakdown and synthesis, respectively, coupled with histological images and functional phenotyping optimize chances for a definitive OA prognostic panel (21). Due to the interconnectivity of periarticular tissues, we agree with Henrotin and others, and further suggest that integrative analyses of signatures from cartilage and surrounding tissues are needed to understand the complex interrelationships at play. For example, it appears that signaling molecules from skeletal muscle can drastically influence the health of bone and its surrounding structures (25, 26). Furthermore, it appears that increased skeletal muscle strength surrounding the diseased joint can mitigate OA symptoms (27, 28). Moving forward, to better understand the etiology of OA and how best to overcome it, more well-controlled integrative molecular mapping and signaling studies are a high research priority.

### OA Treatments

Many treatments exist for OA, and major guidelines have recently been published providing recommendations for their usage (29–31). Most currently available treatments primarily target symptom management as opposed to disease pathobiology. There are currently no therapeutic agents that reverse or halt OA progression, resulting in continued disease progression in millions of patients with OA (30–32). A summary of the treatments covered in this review can be found in Table 1.

**Table 1:** Summary of discussed osteoarthritis treatments and their proposed effects.

**Treatment**	**Effect**
**Pharmacologics**	
Non-steroidal anti-inflammatory drugs	Anti-inflammatory and analgesic (33)
Intra-articular glucocorticoids	Anti-inflammatory and analgesic (33, 34)
Opioids	Analgesic (35)
**Nutraceuticals**	
Blueberries	Analgesic (36, 37)
Montmorency Cherry Juice	Anti-inflammatory and analgesic (37–39)
Glucosamine and/or Chondroitin	Anti-inflammatory and analgesic (40–44)
Curcumin/turmeric	Anti-inflammatory and analgesic (45, 46)
**Biologics**	
Platelet rich plasma	Anti-inflammatory (47–52)
Stem cell therapies	Potential tissue regrowth and analgesic (53–55)
Nerve growth factor antibodies	Anti-inflammatory and analgesic (56)
Fibroblast growth factor 18	Chondroprotective (57)
Interleukin 1 alpha/beta antagonists	Anti-inflammatory (58)
**Exercise**	
Aquatic exercise	Analgesic and improved physical function (59–62)
Aerobic exercise (weight bearing)	Analgesic and improved physical function (63, 64)
Resistance exercise	Analgesic and improved physical function (64–66)
Blood flow restriction (BFR) exercise	Analgesic and improved physical function (67, 68)

#### Pharmacologics

Drugs such as non-steroidal anti-inflammatory drugs (NSAIDs) and intra-articular glucocorticoids are widely used to manage inflammation and pain, but they are best for short-term use (33) as long-term use can lead to significant toxicities (33). Further, the effectiveness of glucocorticoids diminishes over time, which limits their utility for long-term use in OA (33). Long-term repeated intra-articular glucocorticoid injections may be detrimental to cartilage volume in some OA patient populations due to degradative effects on musculoskeletal tissues (34).

#### Nutraceuticals

Due to the lack of substantive benefit and risks of chronic opioid use in OA (35), nutraceuticals have come into the fray for improving quality of life. Specifically, blueberries (36), Montmorency Cherry Juice (38), glucosamine and/or chondroitin (40–42), and curcumin/turmeric (45, 46) have been interrogated. Blueberries and Montmorency Cherry Juice possess large anthocyanin contents which have been shown to attenuate the cyclooxygenase II (COX2) pathway potentially leading to a decrease in pain (37, 39). Both treatments improved Western Ontario McMaster Osteoarthritis Index (WOMAC), and Montmorency Cherry Juice improved high sensitivity C-reactive protein (36, 39). However, blueberry use did not improve plasma markers of inflammation (36). Glucosamine also acts as an anti-inflammatory via the COX2 pathway; the data on its effectiveness in OA treatment are mixed (43, 44), but some encouraging data exist for pain relief (41). Recent systematic reviews of curcumin/turmeric extracts or chondroitin suggest modest pain benefits with little to no untoward side effects (42, 45, 46). Overall, it appears nutraceuticals may help improve quality of life for those suffering with OA without the negative side effects of traditional pharmacological agents. However, large-scale, double-blind, randomized controlled trials to evaluate long term effectiveness of nutraceuticals are warranted, particularly in light of the placebo effect often associated with such compounds.

#### Biologics

Recent technological advancements have prompted new treatments for OA such as platelet rich plasma (PRP) (47–50), intra-articular delivery systems (33), stem cell therapies (53–55), and antibodies to nerve growth factor (NGF) (56). There is still much to be learned about these potential therapies, but some initial findings have been purported. PRP is derived from plasma, and through centrifugation, a concentration of platelets can then be reinjected into the diseased body location (47, 51). PRP is suggested to elicit beneficial changes in the OA joint by quenching the pro-inflammatory signaling cascade through the application of activated PRP containing high concentrations of growth factors, fibrin, and other molecules (49, 51, 52). Intra-articular delivery systems often function by modifying pro-inflammatory molecules through pharmacologic agents such as NSAIDs or steroids bound to particles that can be injected into the diseased joint (33). While these treatments may have promise, the contraindications of frequent injections or possible infection must be considered (33), and as with nutraceuticals, large-scale, double-blind, randomized controlled trials are limited (69, 70).

With stem cell therapies there is vast diversity in the cell source and application method within the joint (55). This heterogeneity accompanied with different study designs and an inability to fully quantify treatment effectiveness makes it difficult to determine the breadth of effectiveness of cell-based treatments (55). However, some recent well-controlled clinical trials (54) illustrate promising improvements in functional/pain scales such as WOMAC following the implantation of autologous bone marrow-derived mesenchymal stromal cells in patients with knee OA. The potential for stem cell therapies is immense as degenerated tissue may be regrown; however, sufficient data are lacking as to the long-term benefits and risks.

Another innovative treatment for patients with OA is the use of antibodies to block the pain signaling of NGF (56). NGF stimulates increased inflammation, and secretion of calcitonin gene-related peptide and substance P which relay pain signals through the nociceptor neuron (56). Antibody based inhibitors of NGF and/or its receptors have proven effective in mitigating pain and improving function in patients with OA, but at higher doses may be detrimental to disease progression (56). Several developmental programs for NGF therapies are on hold, but some are continuing. Therefore, it is vital to determine the mechanisms by which anti-NGF antibodies may negatively influence OA joint health at higher doses if it is to be widely used for pain management and quality of life improvement in OA. New data are emerging for additional biologic treatments including FGF18 which may be chondroprotective (57) and IL-1α/ß antagonists which may act to quench inflammatory signaling in OA (58).

#### Exercise

As previously mentioned, a viable treatment option to mitigate OA progression is exercise (9, 10). This should come as no surprise as exercise has been shown to elicit numerous signaling changes to the extent that it has been described as the “real polypill” (71). Numerous reviews, meta-analyses and primary studies have been conducted since 2016 interrogating the influence of various exercise modalities and physical activity on OA (59–61, 63–67, 72–90), and the American College of Rheumatology/Arthritis Foundation strongly recommend exercise for OA treatment (29). While strongly recommended, the guidelines for exercise prescription lack specificity due to an underabundance of data (29). Furthermore, vast differences in exercise modality and prescription make comparisons difficult across studies.

**Figure 1:**
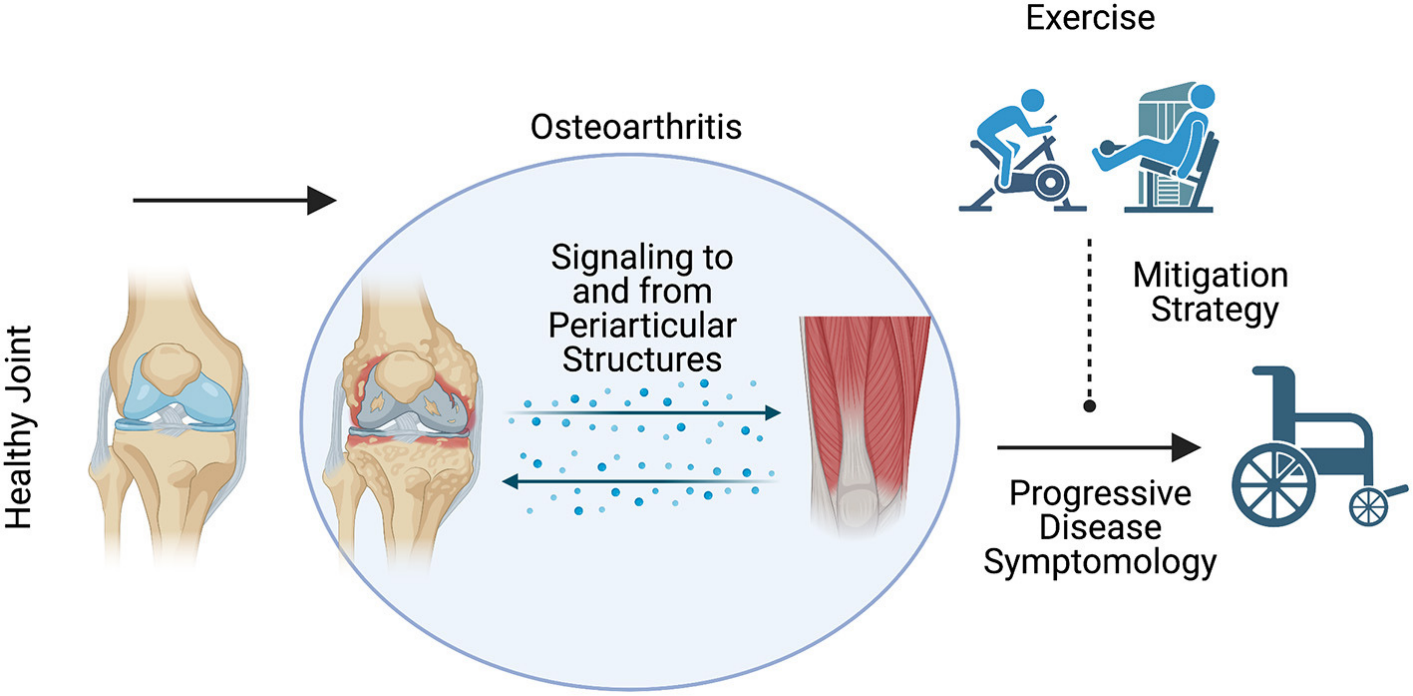
Schematic representation of the expected influence of exercise on osteoarthritis disease symptomology. Exercise may be a viable means to mitigate the deleterious symptoms associated with osteoarthritis disease progression.

For instance, water based exercise training is a common modality as it minimizes the weight bearing load upon the joint and can minimize pain (62); however, the data concerning its effectiveness appear mixed and are often muddled by the inclusion of multiple exercise modalities in and out of the water (59–61). Further, the efficacy of other exercise modalities depends on the outcome of interest (64). Land-based aerobic exercise such as self-selected running does not appear to promote radiographically assessed joint degradation (63) while improving pain and physical function (64). Resistance training appears to elicit similar outcomes (64), and even when performed explosively appears to be a safe means of training for patients with OA (65). While traditional resistance training appears safe, resistance training protocols such as blood flow restriction (BFR) may also be viable while minimizing pain during exercise. Blood flow restriction is a resistance training method that utilizes low loads while applying an occlusion cuff proximal to the muscles being contracted (68). In patients with OA, BFR elicited similar gains in strength, quadriceps cross-sectional area, and timed stand repetitions compared to high intensity resistance training (67). Overall, the data seem to support beneficial outcomes associated with exercise (Figure 1), but the specific benefits such as pain or strength improvement seem to be dictated by exercise programming. Recently, a systematic review by Turner et al. described moderate to large effect sizes for pain and functional outcomes associated with approximately 24 sessions across 8–12 weeks of resistance training (66). However, the need for comprehensive studies interrogating optimal volume, intensity, frequency, exercise selection and mode of delivery are needed to continue the strides made toward best practices for exercise training to treat OA. Adherence to exercise as a long-term OA treatment modality is another major challenge (91). Future studies of behavioral interventions to increase long-term adherence to exercise are also needed.

### End-Stage OA and Rehabilitation Following Total Joint Arthroplasty

End-stage OA is often symptomatically described as a severe decline in the functionality of the affected joint (92), commonly accompanied by severe pain (93), and in some instances presents as bone on bone contact radiographically (94). In end-stage knee OA, elevated pro-inflammatory markers such as IL-6 in synovial fluid, have been associated with pain, joint swelling, and synovitis (95). Furthermore, ADAMTS enzymes have been implicated in cartilage degradation (23), with ADAMTS-4 and−5 specifically linked to knee OA (96). Interestingly, end-stage OA of the hip and knee may differ mechanistically as recent evidence purports decreased ADAMTS-4 and−5 in patients with hip OA (97), contrary to what has been shown in knee OA (96). Furthermore, work from our group has also described heterogeneity among patients with the same diseased joint, end-stage hip OA. This work reported that a subset of patients with end-stage hip OA possess profound pro-inflammatory gene expression within the skeletal muscle surrounding the diseased joint, described as muscle inflammation susceptibility (16). Taken together there appears to be a robust inflammatory cascade in the tissues comprising a joint in the end-stage of OA. This results in pain, functional deficits, and decreased quality of life that often drive patients to elect TJA as a surgical treatment.

TJA is a common procedure that provides many patients with relief from the moderate to severe symptoms of end-stage OA (98). Unfortunately, not all patients have the same beneficial experience, as many suffer a persistent pain and mobility impairment following the procedure (99, 100). Some data suggest pre-surgical frailty leads to higher incidence of poor post-surgical outcomes (101). This is further supported by data illustrating decreased muscle integrity at the whole-muscle and histological levels (102). Work from our research program furthers the notion that overt pro-inflammatory signaling (muscle inflammation susceptibility) in skeletal muscle surrounding the diseased joint is associated with diminished rates of muscle protein synthesis (16). To counteract the poor physiological characteristics of peri-articular muscles leading up to joint replacement, and to diminish the effects of trauma associated with joint repair, many studies have aimed to optimize the rehabilitation process. Unfortunately, there has yet to be a standardized prehabilitation (30) or post-operative rehabilitation protocol within the field (103, 104). This may be in part due to the heterogeneity in recovery outcomes (100, 105), but the consistent lack of appropriate rehabilitation dosage for recovery cannot be ignored (104). Therefore, it is crucial to establish rehabilitation recommendations that effectively address functional limitations following TJA.

A wide range of rehabilitation approaches, perhaps best characterized as “usual care,” has yielded varying levels of efficacy post-TJA. However, many groups including our own hypothesize that progressive and intensive resistance training may be a key ingredient to optimize patient outcomes after joint replacement. When compared to usual care, progressive resistance training (PRT) improves functional measures such as leg press strength without increasing pain (106). Furthermore, a recent meta-analysis indicated progressive resistance training significantly enhances knee extension strength on the surgical limb (107). Results of studies using other functional outcomes such as 6 min walk test (6 MWT) appear to be inconsistent. Some authors purport no change following PRT (106, 107), while others illustrate improvements, but suffer from lack of comparison to a usual care group (108, 109). In general, it appears that quadriceps strength is correlated with the 6 MWT (110), but more data are needed. Encouragingly, supervised PRT has been shown to overcome inflammatory and disuse associated conditions such as aging or bedrest (111–114); therefore, it is logical to posit that provided with appropriate stimulus, treatment of OA with PRT would be effective. However, there are insufficient data and great variability in rehabilitation programming with regard to post-TJA and exercise training for rehabilitation (107, 115, 116). The field remains in a state of need for well-controlled, randomized clinical trials that assess the functional impact of PRT vs. usual care and shed light on the mechanistic underpinnings differentiating these widely divergent rehabilitation approaches. Work addressing these issues would provide a needed basis for optimized exercise programming following TJA, potentially lead to pharmacological adjuvants, and improve the likelihood of successful recovery following surgery.

## Conclusions

OA remains a complex disease state that is not fully understood. However, remarkable strides have been made recently that aim to promote improved functionality and quality of life for those living with the disease. These advancements aim to potentially prevent or at least prolong the time before patients undergo more invasive procedures such as TJA. While TJA appears effective for many, little is known about why some patients continue to suffer from severe symptoms following the procedure, with even less known about how to optimally alleviate these problems. Exercise in particular appears to beneficial as a treatment for pain management and functional improvements with OA, and progressive resistance training may be necessary to optimally recover following TJA. However, many uncertainties persist within the field which limit the impact of exercise as an effective treatment or therapeutic option. To overcome this, comprehensive large-scale rehabilitation clinical trials are needed to better inform practitioners moving forward with rehabilitative care.

## Author Contributions

DD co-conceptualized the manuscript, performed literature search, and wrote the manuscript. MB co-conceptualized the manuscript, edited the manuscript, and provided additional literature for inclusion. JM, RS, AF, SB, and JS edited the manuscript and provided additional literature for inclusion. All authors contributed to manuscript revision, read, and approved the submitted version.

## Conflict of Interest

JS has received consultant fees from Crealta/Horizon, Medisys, Fidia, PK Med, Two labs Inc, Adept Field Solutions, Clinical Care options, Clearview healthcare partners, Putnam associates, Focus forward, Navigant consulting, Spherix, MedIQ, Jupiter Life Science, UBM LLC, Trio Health, Medscape, WebMD, and Practice Point communications; and the National Institutes of Health and the American College of Rheumatology. JS owns stock options in TPT Global Tech, Vaxart pharmaceuticals and Charlotte's Web Holdings, Inc. JS previously owned stock options in Amarin, Viking and Moderna pharmaceuticals. JS is on the speaker's bureau of Simply Speaking. JS is a member of the executive of Outcomes Measures in Rheumatology (OMERACT), an organization that develops outcome measures in rheumatology and receives arms-length funding from 8 companies. JS serves on the FDA Arthritis Advisory Committee. JS is the chair of the Veterans Affairs Rheumatology Field Advisory Committee. JS is the editor and the Director of the University of Alabama at Birmingham (UAB) Cochrane Musculoskeletal Group Satellite Center on Network Meta-analysis. JS previously served as a member of the following committees: member, the American College of Rheumatology's (ACR) Annual Meeting Planning Committee (AMPC) and Quality of Care Committees, the Chair of the ACR Meet-the-Professor, Workshop and Study Group Subcommittee and the co-chair of the ACR Criteria and Response Criteria subcommittee. The remaining authors declare that the research was conducted in the absence of any commercial or financial relationships that could be construed as a potential conflict of interest.

## Publisher's Note

All claims expressed in this article are solely those of the authors and do not necessarily represent those of their affiliated organizations, or those of the publisher, the editors and the reviewers. Any product that may be evaluated in this article, or claim that may be made by its manufacturer, is not guaranteed or endorsed by the publisher.
